# Acute Stress Attenuates Hepatic Ischemia–Reperfusion Injury via Hypothalamic CRH Neuron‐Induced HPA Axis Activation

**DOI:** 10.1002/cns.70749

**Published:** 2026-01-14

**Authors:** Xiaoqi Lin, Dan Yang, Shuyang Wang, Baoshan Wang, Ling Zhu, Yanyu Zhou, Yifei Zhou, Song Zhang, Qionghui Zhan, Yingfu Jiao, Weifeng Yu, Liqun Yang, Po Gao

**Affiliations:** ^1^ Department of Anesthesiology, Renji Hospital Shanghai Jiao Tong University School of Medicine Shanghai China; ^2^ Key Laboratory of Anesthesiology (Shanghai Jiao Tong University) Ministry of Education Shanghai China; ^3^ Department of Pain The First Affiliated Hospital of Wenzhou Medical University Wenzhou Zhejiang China; ^4^ Oujiang Laboratory (Zhejiang Lab for Regenerative Medicine, Vision and Brain Health) Wenzhou Medical University Wenzhou Zhejiang China; ^5^ Department of Anesthesiology The Fourth Affiliated Hospital of Anhui Medical University Chaohu Anhui China

**Keywords:** acute stress, CRH neurons, hepatic ischemia–reperfusion injury, HPA axis, hypothalamic paraventricular nucleus

## Abstract

**Background:**

Hepatic ischemia–reperfusion injury (HIRI) is a pathologic process commonly encountered during liver surgery, which seriously threatens patient prognosis. Currently, effective interventions or preventive measures are still lacking. Notably, patients with liver disease commonly experience brief acute stress prior to surgery; however, the impact of acute stress on HIRI remains unclear.

**Methods:**

A 30‐min restraint stress was used to simulate acute restraint stress (ARS). Hematoxylin–eosin staining and ELISA were employed to assess HIRI. Immunofluorescence staining and electrophysiology were applied to evaluate neuronal activation. Chemogenetic manipulation was utilized to verify the role of corticotropin‐releasing hormone (CRH) neurons in the hypothalamic paraventricular nucleus (PVN) in ARS‐mediated attenuation of HIRI.

**Results:**

The results showed that ARS significantly ameliorated liver injury, reduced the liver enzyme levels (ALT and AST), and down‐regulated the inflammatory factors expression in HIRI mice. Furthermore, we found that ARS alleviated HIRI by activating the hypothalamic–pituitary–adrenal (HPA) axis to release corticosterone, rather than through the sympathetic nervous system. PVN^CRH^ represented a critical subpopulation responding to ARS. Chemogenetic activation of PVN^CRH^ neurons mimicked the protective effect of ARS against HIRI, whereas chemogenetic inhibition of these neurons abolished this protection.

**Conclusion:**

Our findings demonstrate that PVN^CRH^ neurons mediate the protective effect of ARS against HIRI by activating the HPA axis to release corticosterone. This work may provide key insights for developing perioperative strategies to prevent HIRI.

## Graphical abstract

This study demonstrates the protective effect of acute restraint stress (ARS) against hepatic ischemia–reperfusion injury (HIRI) in mice and investigates its specific neuroendocrine mechanisms. During ARS, excitability of PVN^CRH^ neurons significantly increases, which drives HPA axis to release corticosterone, thereby reducing hepatic inflammation and ultimately alleviating HIRI.

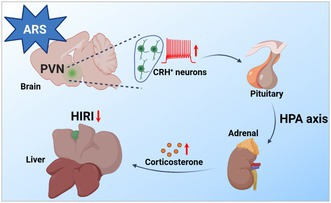

## 1 Introduction

Hepatic ischemia–reperfusion injury (HIRI) is an inevitable complication of liver surgery, especially liver transplantation [1]. HIRI is closely associated with a wide range of complications, such as hepatic failure, early graft dysfunction, and acute or chronic rejection, which can further progress to systemic inflammatory response syndrome (SIRS) or multiple organ failure (MODS), which is a serious threat to patients' lives [2, 3]. However, traditional therapies, including pharmacological interventions, preconditioning/postconditioning, and machine perfusion, suffer from poor clinical efficacy, low maneuverability, and lack of sophisticated equipment, which hinder the treatment of HIRI and the development of liver surgery [1, 4].

During the perioperative period, patients with liver disease often experience varying degrees of stress [5, 6]. Acute or mild stress often exerts a protective effect on the organism [7]. Liu and colleagues' study shows that environmental eustress promotes regeneration via the sympathetic–immune axis by enhancing the intrahepatic ILC1–IL‐22–STAT3 signaling pathway [8]. Short‐term stress preconditioning mobilizes central noradrenergic circuits and augments sympathetic outflow to reprogram innate immune responses, thereby mitigating testicular ischemia–reperfusion injury [9]. Clinically, multimodal prehabilitation integrating exercise, nutrition, and psychological interventions—conceptually aligned with controlled eustress—reduces postoperative complications, shortens hospital stay, and improves recovery [10]. Conversely, chronic or severe stress most frequently impairs the function of peripheral organs [11, 12, 13]. Our previous study [14] demonstrated that chronic stress markedly suppresses liver regeneration following partial hepatectomy. It is noteworthy that research on the impact of preoperative acute stress on HIRI and the underlying neural regulatory mechanisms is somewhat lacking.

The central nervous system (CNS) is the pivotal organ in the stress response. In recent years, the role of the CNS in regulating peripheral organ function in response to stress has garnered widespread attention. The peripheral organs such as the stomach [11], spleen [15], bone [13], and gut [16] have been reported to be regulated by the CNS in response to stress. The hypothalamus is the core functional nucleus mediating stress effects, which can regulate peripheral organ functions downstream by activating the HPA axis or the autonomic nervous system (particularly the sympathetic nervous system) [17, 18, 19, 20]. Yet, it remains unclear which brain nuclei and neuronal subpopulations mediate the regulation of hepatic homeostasis in response to acute stress.

In this study, we explored the effects of acute stress on HIRI and its underlying mechanisms. Our data indicate that acute stress exerts a protective effect against HIRI by activating CRH neurons in the PVN, and this protection is mediated through the HPA axis rather than the sympathetic nervous system (SNS) to alleviate liver inflammation. Together, these findings offer valuable opportunities for improving outcomes in patients undergoing liver surgery.

## 2 Materials and Methods

### 2.1 Animals

Adult male *Crh‐Cre* mice and Ai47 mice were obtained from Professor Ji Hu's Laboratory (ShanghaiTech University). Adult male wild type C57BL/6 mice were purchased from Shanghai Jiesijie Laboratory Animal Co. Ltd. All animals were housed in a temperature‐controlled room (22°C–25°C) under a 12 h light/dark cycle with food and water available ad libitum. Mice were randomly assigned to experimental groups. The investigator was blinded to group identity during the experiment and quantitative analyses. All animal procedures were approved by the Ethics Committee for Experimental Use of Animals of Shanghai Jiao Tong University School of Medicine.

### 2.2 Acute Restraint Stress Model

Acute restraint stress (ARS) model was established as previously described [21]. Briefly, mice were placed in a 50‐mL conical tube with a venting hole at the tip for 30 min, and cotton was stuffed into the empty part of the tube. Continuous monitoring was conducted to ensure mice could not move freely while respiratory function remained unaffected. After each trial, centrifuge tubes were cleaned with 75% alcohol.

### 2.3 The Open Field Test

The open field test (OFT) was carried out to assess the anxiety‐like behavior of mice. The open field chamber is an opaque plastic box (40 cm × 40 cm × 40 cm) placed in a sound‐attenuated room. A 20 cm × 20 cm center square was defined as the central area. Before each test, mice are transferred to the test room to acclimate to the environment 1 h in advance. Mice were placed into the center individually, and their spontaneous behavior was monitored for 10 min. The time spent in center and total distance were recorded and analyzed throughout the experiment. After each trial, the chamber was cleaned with 75% ethanol.

### 2.4 Hepatic Ischemia–Reperfusion Injury Model

Hepatic ischemia–reperfusion injury model was established as previously described [22]. Male mice (8–10 weeks old) were anesthetized with pentobarbitone (50 mg/kg, i.p.), and a midline incision on the abdomen was made to expose the liver. An atraumatic clip was used to block the liver blood supply of the left lateral lobe and the median lobe for 45 min, and then the clip was removed for 6 h reperfusion, while the sham group underwent the same surgery without vascular occlusion.

After 45 min ischemia and 6 h reperfusion, liver samples and serum samples were collected for examination. Blood samples were centrifuged to obtain serum. To evaluate the extent of hepatocyte damage, the levels of serum aminotransferases (ALT and AST) were measured using an automated chemical analyzer (Rayto Life and Analytical Sciences Co. Ltd. Chemray 800, Shenzhen, China).

### 2.5 Hematoxylin–Eosin (H&E) Staining and Terminal dUTP Nick‐End Labeling (TUNEL) Staining of Liver

Histopathological damage was examined by H&E staining. Liver samples were fixed in 4% formaldehyde, embedded in paraffin, and then sectioned at 5 μm. After dewaxing, liver sections were stained with hematoxylin and eosin. The extent of liver injury was assessed using a blinded method. Liver sections were observed under an optical microscope, and four randomly selected areas from each sample were scored according to the Sukuzi criteria.

Liver tissue apoptosis was detected with TUNEL assay. Liver sections were obtained as described above, and then TUNEL staining was performed using an in situ cell death detection kit according to the manufacturer's instructions. Apoptotic cells (where the nuclei of positive cells were stained brown) were observed and photographed under an optical microscope.

### 2.6 Quantitative Polymerase Chain Reaction (qPCR)

Total RNA was isolated from mouse liver tissue using the Tissue RNA Purification Kit (EZBioscience, China) in accordance with the manufacturer's guidelines. The extracted RNA was converted to first‐strand cDNA with the 4× EZscript Reverse Transcription Mix II for qPCR (EZBioscience, China). Quantitative real‐time PCR was carried out on a dedicated real‐time platform employing the 2× SYBR Green qPCR Master Mix (EZBioscience, China). GAPDH was used as the housekeeping control, and relative transcript levels were determined using the $2^{-\Delta \Delta C_{t}}$ approach. Primer sequences are provided in Table S1.

### 2.7 Enzyme‐Linked Immunosorbent Assay (ELISA)

The level of norepinephrine (U96‐3531E, YOBIBIO) in liver tissue and levels of corticosterone (U96‐1106E, YOBIBIO), TNF‐α (GEM0004‐96 T, Servicebio), and IL‐1β (GEM0002‐96 T, Servicebio) in liver tissue and serum were detected by ELISA kits according to the manufacturers' instructions.

### 2.8 Viral Injection and Chemogenetic Modulation

Mice (8–10 weeks old) were anesthetized with pentobarbitone (50 mg/kg, i.p.) and secured in a stereotactic frame (RWD Instruments). A midline scalp incision was made and a hole was drilled on the skull to allow the passage of a glass pipette filled with the virus. Thereafter, the virus was injected into PVN (coordinates from Bregma: AP: −0.83 mm, ML: ±0.22 mm, DV: −5.00 mm) at a speed of 50 nL/min, allowing an additional 10 min for viral particles to diffuse before the pipette was slowly withdrawn. The virus, including AAV2/9‐EF1α‐DIO‐mCherry‐WPRE‐hGH‐pA, AAV2/9‐EF1α‐DIO‐hM3D(Gq)‐mCherry‐WPRE‐hGH‐pA, and AAV2/9‐EF1α‐DIO‐hM4D(Gi)‐mCherry‐WPRE‐hGH‐pA, was purchased from BrainVTA (China).

Chemogenetic manipulation of CRH neurons was carried out using the DREADD ligand clozapine‐N‐oxide (CNO). Three weeks after receiving the viral injection, mice were administered CNO (1 mg/kg, i.p.) 30 min before surgery or restraint procedures.

### 2.9 Immunofluorescence (IF) Staining

Brain samples were fixed in 4% formaldehyde for at least 6 h, dehydrated by 30% sucrose and then cut into 30‐μm‐thick sections. The antigens were retrieved by incubation overnight at 4°C with the following primary antibodies: c‐Fos (1:1000, ab208942, Abcam), AVP (1:1000, ab213708, Abcam), OXT (1:1000, ab212193, Abcam), nNOS (1:800, ab76067, Abcam), GPER (1:1000, ab39742, Abcam) and TH (1:1000, ab134461, Abcam). The sections were then incubated with fluorescence conjugated secondary antibodies (1:1000, Abcam) at room temperature for 1.5 h. The secondary antibodies used in this study were as follows: Donkey anti‐Mouse IgG H&L (Alexa Fluor 488, ab150105), Goat anti‐Rabbit IgG H&L (Alexa Fluor 594, ab150080), and Goat anti‐Chicken IgY H&L (Alexa Fluor 594, ab150176).

### 2.10 Brain Slice Electrophysiology

#### 2.10.1 Slice Preparation

PVN slice preparation was performed as described previously [14]. Briefly, male *Crh‐Cre::Ai47* mice were deeply anesthetized with pentobarbital sodium (0.5% w/v, i.p.) and intracardially perfused with ~20 mL oxygenated modified NMDG artificial cerebrospinal fluid (NMDG ACSF) containing the following (in mM): 92 N‐methyl‐d‐glucamine (NMDG), 2.5 KCl, 30 NaHCO_3_, 25 Glucose, 1.25 NaH_2_PO_4_, 20 HEPES, 3 Na‐pyruvate, 2 Thiourea, 5 Na‐ascorbate, 0.5 CaCl_2_ and 10 MgSO_4_. The pH of NMDG ACSF was adjusted to 7.3–7.4 with concentrated HCl. Coronal slices (180 μm) that contained the PVN were sectioned at 0.2 mm/s using a vibrating microtome (VT1200s, Leica). The brain slices were initially incubated in NMDG ACSF for 10 min at 32°C and then transferred to HEPES ACSF containing the following (in mM): 92 NaCl, 2.5 KCl, 30 NaHCO_3_, 25 Glucose, 1.25 NaH_2_PO_4_, 20 HEPES, 2 Thiourea, 3 Na‐pyruvate, 5 Na‐ascorbate, 2 CaCl_2_ and 2 MgSO_4_ for at least 1 h at 25°C. Subsequently, the PVN slices were transferred to a recording chamber for electrophysiological experiments and were continuously perfused with standard ACSF containing the following (in mM): 125 NaCl, 2.5 KCl, 2 CaCl_2_, 25 NaHCO_3_, 1 MgCl_2_, 12.5 glucose and 1.25 NaH_2_PO_4_ at 3 mL/min. The experimenters were blinded to group identity during all recordings and data analysis.

#### 2.10.2 Whole‐Cell Patch‐Clamp Recording

PVN neurons in the slice were visualized using a × 40 water‐immersion objective on an upright microscope (BX51WI, Olympus) equipped with infrared differential interference contrast (IR‐DIC) and an infrared camera connected to a video monitor. Whole‐cell patch‐clamp recording was obtained from visually identified PVN neurons. The recording pipettes (6–7 MΩ) were pulled from borosilicate capillaries using a horizontal micropipette puller (P‐1000, Sutter Instrument) and filled with the following internal solution (120 mM K‐gluconate, 10 mM KCL, 5 mM NaCl, 2 mM MgCl_2_·6H_2_O, 1 mM CaCl_2_·2H_2_O, 10 mM HEPES, 11 mM EGTA, 1 mM Li‐GTP, and 2 mM Mg‐ATP, pH was adjusted to 7.3–7.4 with Tris‐base). A 2‐min equilibration period was allowed after whole‐cell access was established to reach steady state. The resting membrane potential was continuously recorded with I = 0 current‐clamp mode. To measure excitability of PVN neurons, the number of action potentials (APs) induced by a series of 500 ms hyper‐ and depolarizing current injections in 10 pA steps (ranging from −40 to 100 pA) was recorded. The membrane potential was held at −70 mV for recording spontaneous excitatory postsynaptic currents (sEPSCs) in voltage‐clamp mode. Membrane voltage and current were amplified using a MultiClamp 700B amplifier (Molecular Devices, USA), filtered at 2 kHz, and digitized at 10 kHz. Data were acquired using pClamp 10.7 software (Molecular Devices, USA). Neurons were discarded if the access or input resistance changed by more than 20%.

#### 2.10.3 Biocytin Diffusion and Post Hoc Immunofluorescence Staining

To visualize the recorded neurons, biocytin was dissolved in the intracellular solution. After recording from the PVN slices, the whole‐cell mode was maintained for at least 5 min to allow biocytin to diffuse from the electrode into the recorded cell. The brain slices were then fixed in 4% PFA for 2 h, followed by four washes with PBS. Subsequently, the slices were permeabilized in 1% Triton‐X100/PBS for 1 h and incubated with Streptavidin‐Cy3 (1:200, S6402, Sigma) at room temperature for 2 h.

### 2.11 Drug Administration

Corticosterone (CORT) was dissolved in DMSO and diluted with corn oil prior to injection. Mice received an intraperitoneal injection of CORT (1 mg/kg) or corn oil 30 min prior to HIRI surgery.

For chemical denervation of hepatic sympathetic nerve, 6‐Hydroxydopamine hydrobromide (6‐OHDA) (HY‐B1081A, MedChemExpress) was administered by intraperitoneal (i.p.) injection of 100 mg/kg. Mice received an intraperitoneal injection of 6‐OHDA for 5 consecutive days prior to ARS modeling. The drug was dissolved in a saline solution containing 0.1% ascorbic acid.

For mifepristone (HY‐13683, MedChemExpress), a glucocorticoid receptor (GR) antagonist dissolved in DMSO and diluted with corn oil prior to injection, mice received an intraperitoneal injection of mifepristone at the dosage of 10 mg/kg. To block the glucocorticoid receptor, mifepristone was administered 30 min before ARS model.

### 2.12 Statistical Analysis

Statistical analysis was performed using GraphPad Prism 9.0 (GraphPad Software, San Diego, CA, USA). All data are expressed as mean ± SEM. To compare differences between the two groups, the paired or unpaired Student's *t*‐test was used. For multiple group comparisons, one‐way or two‐way ANOVA followed by Sidak's post hoc test was used. A *p* value of < 0.05 was considered significant.

## 3 Results

### 3.1 Acute Stress Attenuates Hepatic Ischemia–Reperfusion Injury

**FIGURE 1:**
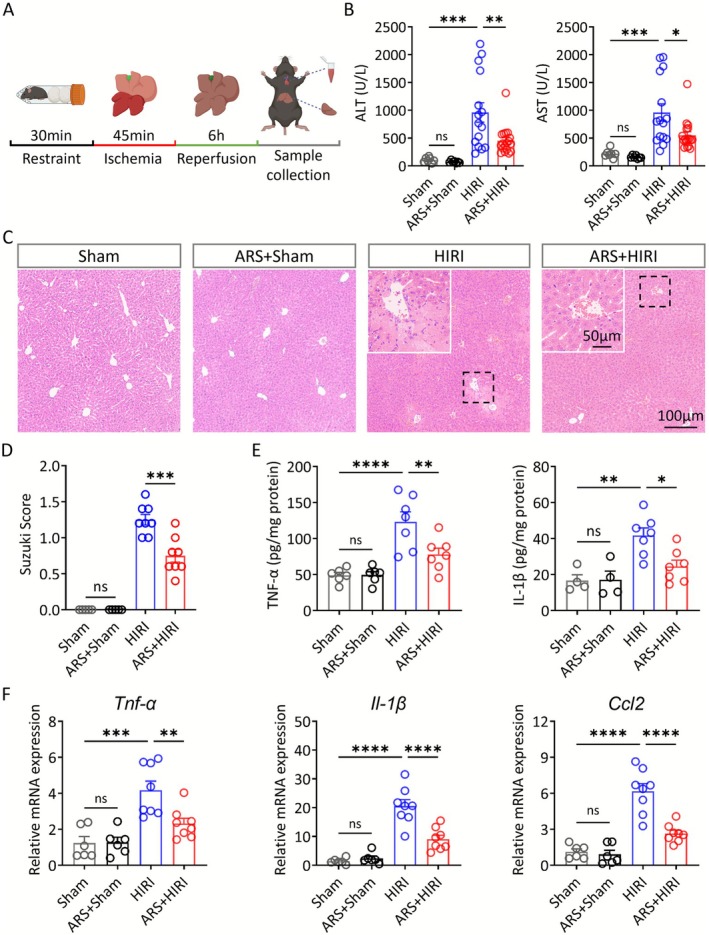
ARS attenuates HIRI. (A) Experimental design. Mice were exposed to 30‐min restraint before hepatic I/R surgery. (B) Serum ALT and AST levels in mice from each group (*n* = 7–18). (C, D) Representative images and Suzuki score of H&E staining of liver tissues from each group (*n* = 6–8). (E) Serum concentrations of TNF‐α and IL‐1β (*n* = 4–7). (F) Relative expression of mRNAs for cytokines of *Tnf‐α*, *Il‐1β*, and *Ccl2* (*n* = 6–8). One‐way ANOVA (B, D, E, and F). ns, not significant; * *p* < 0.05; **p < 0.01; ***p < 0.001; ****p < 0.0001.

We first constructed an acute restraint stress (ARS) model and performed the open‐field test (OFT), which revealed that the 30‐min acute stress did not induce anxiety‐like behavior in mice (Figure S1A,B). To demonstrate the effect of acute stress on HIRI, hepatic I/R surgery was performed on mice after acute stress modeling (Figure 1A). After 45 min of ischemia and 6 h of reperfusion, blood and liver samples were collected and subsequently analyzed for liver function. The results showed that the elevated serum levels of ALT and AST at 6 h after reperfusion were significantly attenuated in ARS mice (Figure 1B). Furthermore, we found that the number of apoptotic cells shown by TUNEL staining was reduced in the ARS + HIRI group compared with the HIRI group (Figure S1C). Meanwhile, we used hematoxylin and eosin (H&E) staining to detect the histological damage level of the liver (Figure 1C). The Suzuki score shows a significant decrease in the ARS + HIRI group compared to that in the HIRI group (Figure 1D).

Complicated immune responses and excessive inflammation are recognized as obligatory processes in the pathology of HIRI [1]. Therefore, we examined the mRNA levels of the classical inflammatory factors, *Tnf‐α*, *Il‐1β*, and *Ccl2*, and the protein expression of TNF‐α and IL‐1β (Figure 1E,F). The results showed that ARS can attenuate the inflammatory response following HIRI.

### 3.2 Corticosterone Mediates the Protective Effect of ARS on HIRI

**FIGURE 2:**
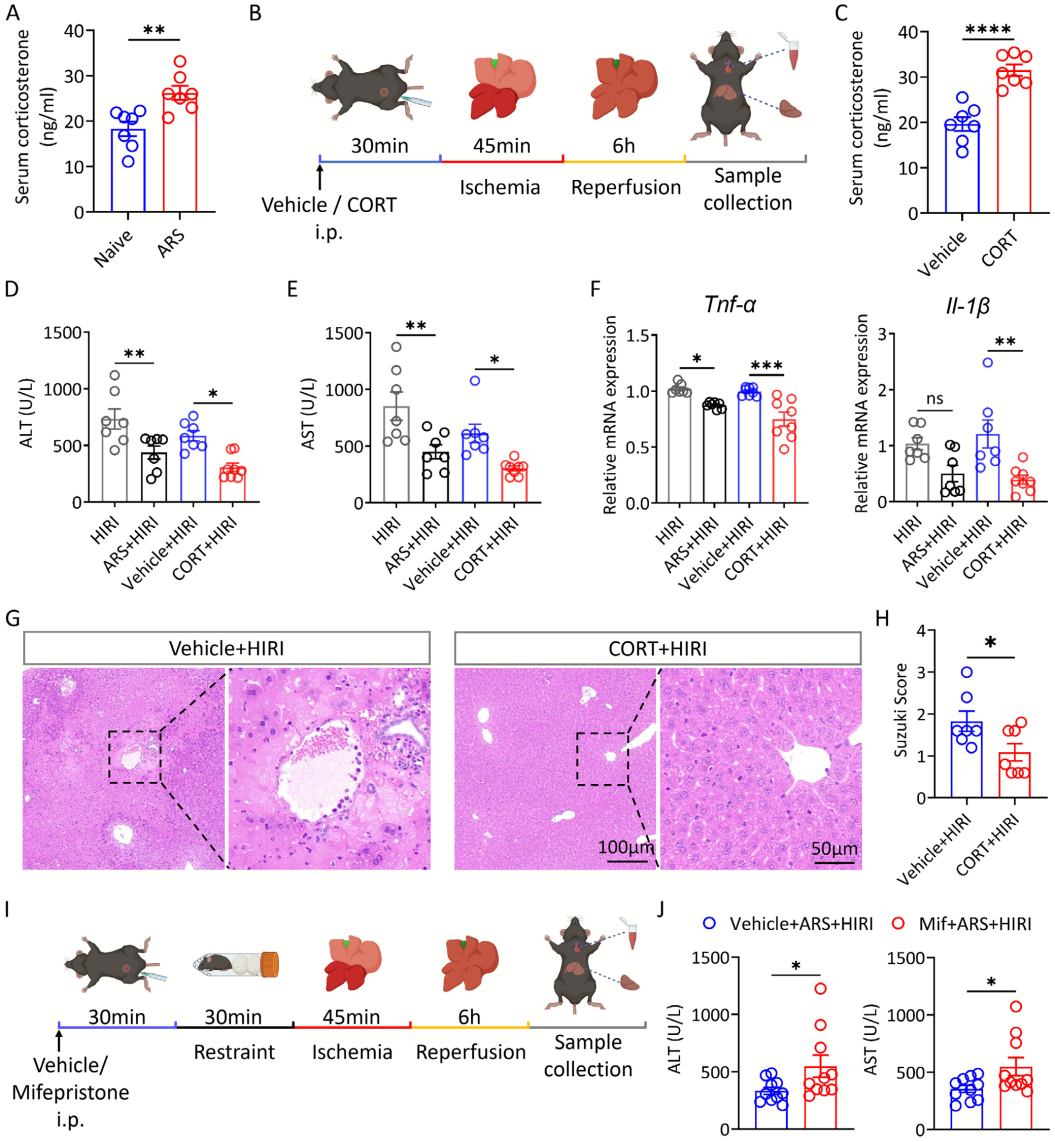
Corticosterone attenuates HIRI. (A) Serum corticosterone levels of Naive and ARS mice (*n* = 7). (B) Experimental outlines for ARS and HIRI modeling after CORT administration. (C) Serum corticosterone levels in mice administered with vehicle and CORT (*n* = 7). (D–E) Serum ALT and AST levels in mice from each group (*n* = 7–8). (F) Relative expression of mRNAs for cytokines of *Tnf‐α* and *Il‐1β* (*n* = 7–8). (G, H) Representative images and Suzuki score of H&E staining of liver tissues from each group (*n* = 7). (I) Experimental outlines for ARS and HIRI modeling after mifepristone administration. (J) Serum ALT and AST levels in mice from each group (*n* = 7–8). Two‐tailed unpaired *t* test (A, C, H and J). One‐way ANOVA (D–F). ns, not significant; * *p* < 0.05; **p < 0.01; ***p < 0.001; ****p < 0.0001.

The activation of the HPA axis is one of the most striking features of stress [23]. We examined the serum corticosterone levels after ARS and found a significant elevation in ARS mice (Figure 2A). To investigate whether the protective effect of ARS on HIRI was mediated by the HPA axis, we simulated ARS by intraperitoneal injection of corticosterone (CORT) and then performed hepatic I/R surgery (Figure 2B,C). We found that CORT treatment remarkably reduced the transaminase levels, the mRNA expression of *Tnf‐α*, *Il‐1β*, and *Ccl2*, apoptosis, and tissue necrosis after hepatic I/R surgery (Figure 2D–H, Figure S2A,B).

Furthermore, we utilized the corticosterone receptor blocker, mifepristone, to validate the role of CORT in regulating HIRI (Figure 2I). We observed that ALT and AST levels were significantly elevated in the Mif + ARS + HIRI group compared to that in the ARS + HIRI group (Figure 2J). HE staining and qPCR results also demonstrated that the levels of tissue damage and inflammation were significantly higher in the Mif + ARS + HIRI group (Figure S2C–E). Taken together, we found that corticosterone levels were elevated during ARS, and the increased corticosterone could alleviate HIRI.

### 3.3 Denervation of Hepatic Sympathetic Nerves Does Not Alter the Protective Effect of ARS on HIRI

It is widely recognized that the liver is primarily innervated by sympathetic nerves [24]. Meanwhile, stress is reported to activate the sympathetic nervous system, and restraint stress could protect the kidney against IR injury via a sympathetic route [7]. Therefore, we aimed to investigate whether the protective effects of acute stress on HIRI are related to hepatic sympathetic nerves. We first examined the norepinephrine (NE) content in the liver of ARS mice and naive mice. However, there was no statistical difference in that between ARS mice and naive mice (Figure S3A). Furthermore, we used 6‐hydroxydopamine (6‐OHDA) neurotoxin to induce chemical denervation of the hepatic sympathetic nerves, and the effectiveness of denervation was evidenced by the reduced NE levels in liver tissues of the 6‐OHDA group of mice compared to the Vehicle group of mice (Figure S3B,C). The results showed that removal of hepatic sympathetic nerves had no significant effect on the levels of liver enzyme and tissue injury in mice subjected to ARS and hepatic I/R surgery (Figure S3D–F). Consistently, there was no statistical difference in the mRNA expression levels of inflammatory factors (Figure S3G).

### 3.4 CRH Neurons in the PVN Are Activated by ARS

**FIGURE 3:**
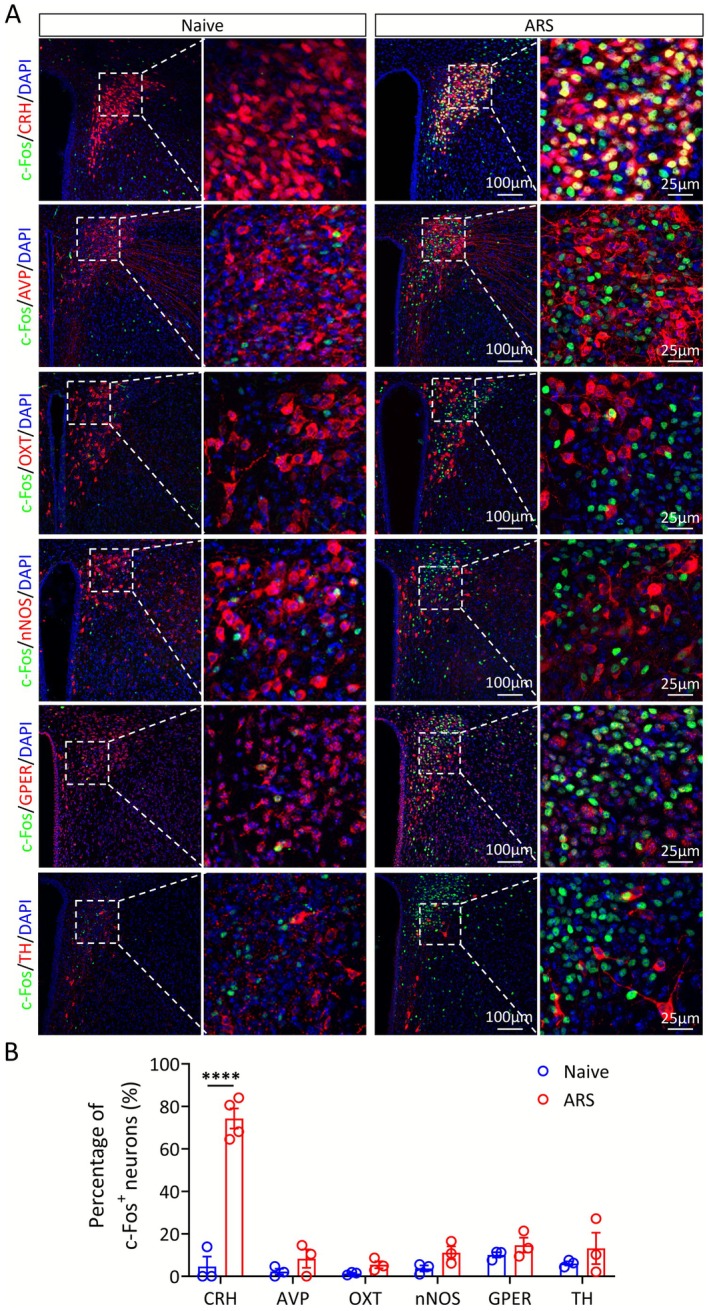
Activation of different neuronal subpopulations in the PVN during ARS. (A) Representative immunofluorescence images showing the expression of c‐Fos (green) and CRH, arginine vasopressin (AVP), oxytocin (OXT), neuronal nitric oxide synthase (nNOS), G protein‐coupled estrogen receptor (GPER) and tyrosine hydroxylase (TH) (red) in PVN neurons of nave and ARS mice. (B) Quantification of c‐Fos expression levels in the PVN of naive and ARS groups (*n* = 3–4). Two‐tailed unpaired *t* test (B). ****p < 0.0001.

To target the stress response upstream of the HPA axis, we turned our attention to changes in the central nervous system. Notably, the paraventricular nucleus of the hypothalamus (PVN) in the brain is exactly the key component and starting node of the HPA axis [18, 25]. Thus, we are very intrigued by the neuronal activation of the PVN after acute stress. As shown by immunofluorescence staining for c‐Fos, the neuronal activity marker, the PVN neurons predominantly activated by ARS were CRH‐positive (Figure 3A,B).

**FIGURE 4:**
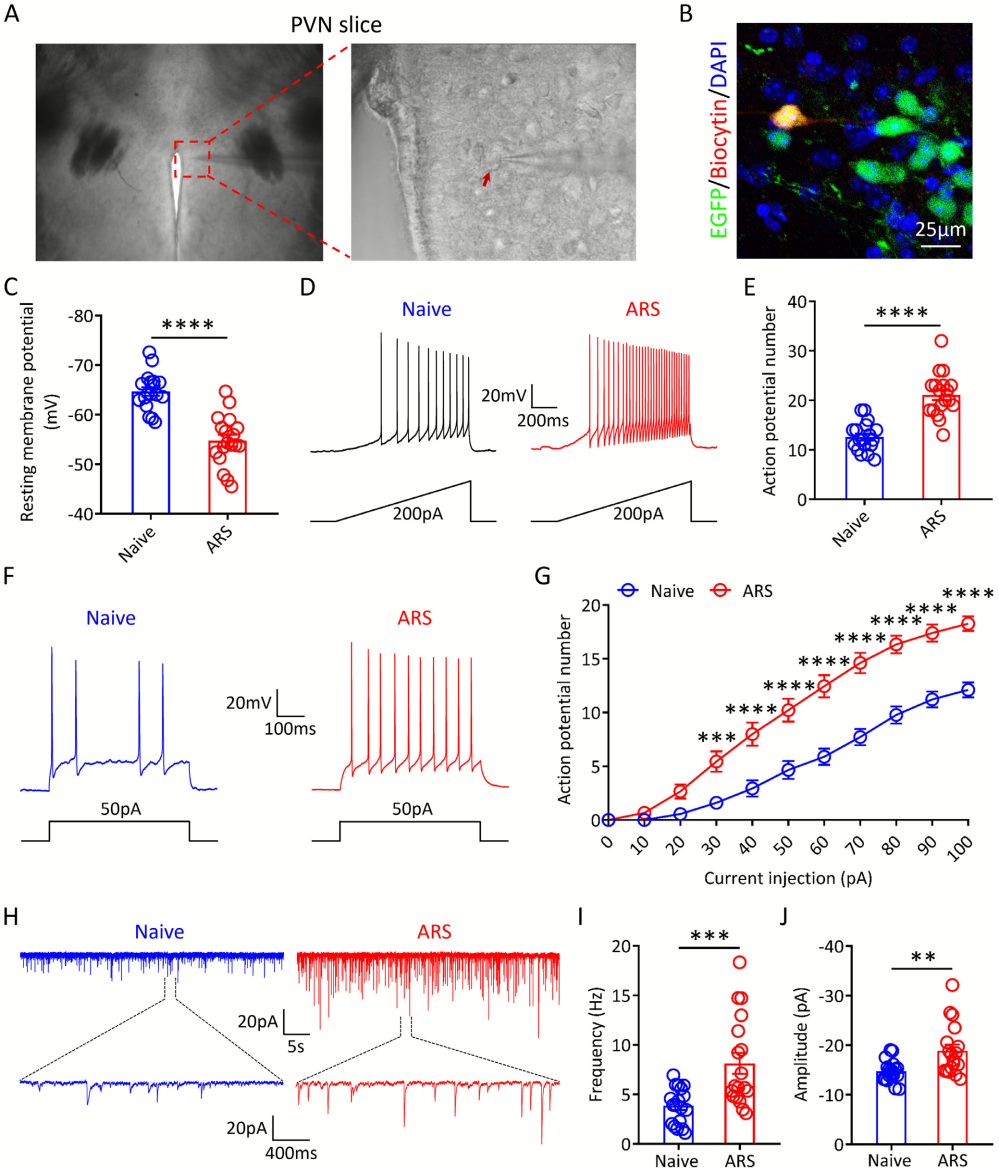
The excitability of PVN^CRH^ neurons is significantly increased during ARS. (A, B) Representative image of whole‐cell recording of PVN neurons and the selected PVN^CRH^ neuron was labeled by biocytin for post hoc identification of the cell identity. (C) Resting membrane potential (RMP) of PVN^CRH^ neurons in the naive and ARS mice (*n* = 18 cells from 3 mice in each group). (D, E) Representative APs of PVN^CRH^ neurons in response to a 200 pA ramp of depolarizing current over a 1000 ms stimulus and statistical analysis of the APs in the naive and ARS groups (*n* = 18 cells from 3 mice in each group). (F) Representative traces of typical AP response to 50 pA depolarizing current injection recorded from the PVN^CRH^ neurons of naive and ARS mice, respectively. (G) Statistical analysis of APs in naive and ARS groups under different current stimulation intensities (*n* = 18 cells from 3 mice in each group). (H–J) Representative sEPSCs traces and statistical analysis of the average frequency and amplitude of sEPSCs of PVN^CRH^ neurons in the naive and ARS mice (*n* = 18 cells from 3 mice in each group). Two‐tailed unpaired *t* test (C, E, I and J). Two‐way ANOVA (G). **p < 0.01; ***p < 0.001; ****p < 0.0001.

To further clarify the effects of ARS on PVN^CRH^ neuron activity, we performed whole‐cell patch‐clamp recordings to assess changes in neuronal excitability. For genetic labeling of CRH‐positive neurons, we crossed *Crh‐Cre* mice with Ai47 (Cre‐dependent EGFP reporter) mice to generate *Crh‐Cre*::Ai47 mice. Using *Crh‐Cre::Ai47* mice, we targeted EGFP‐positive neurons for patch‐clamp recording and verified their CRH‐positive identity via post hoc biocytin labeling (Figure 4A,B). Compared to naive mice, PVN^CRH^ neurons from ARS animals displayed a depolarized resting membrane potential and fired more action potentials (APs) in response to equivalent ramp current stimulation (Figure 4C–E). ARS mice exhibited a lower action potential threshold and fired more APs in response to step‐current injections (Figure S4 and Figure 4F,G). Moreover, both the frequency and amplitude of sEPSCs were markedly enhanced in ARS mice relative to naive controls (Figure 4H–J). Collectively, these findings indicate that ARS significantly enhances both the intrinsic excitability and synaptic transmission efficiency of PVN^CRH^ neurons.

### 3.5 Chemogenetic Activation of the PVN^CRH^ Neurons Triggers the Protective Effect on HIRI

**FIGURE 5:**
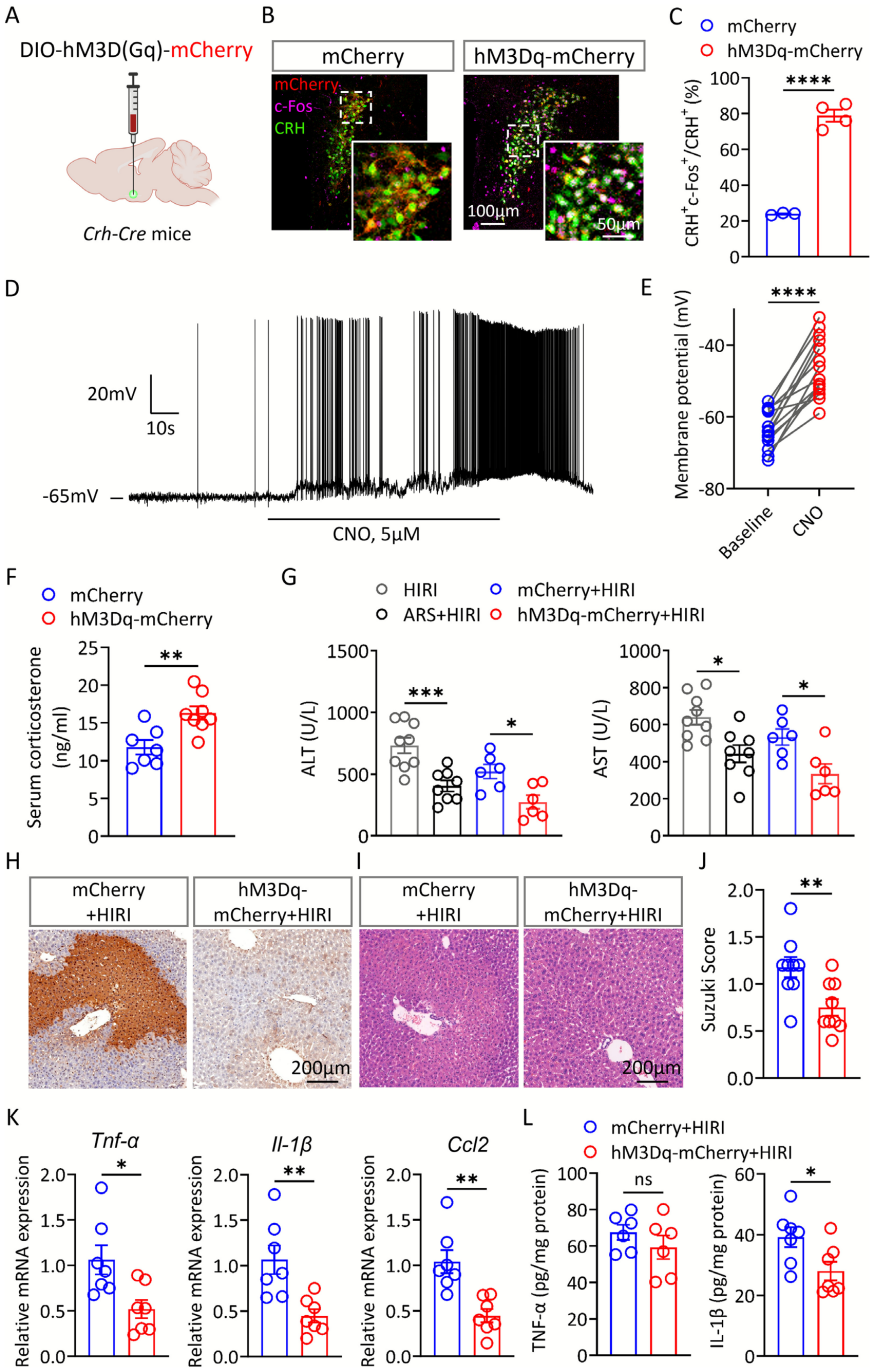
Chemogenetic activation of the PVN^CRH^ neurons triggers the protective effect on HIRI. (A) Paradigm of chemogenetic activation of PVN^CRH^ neurons. (B, C) Representative images showing the expression of mCherry or hM3Dq‐mCherry and c‐Fos in the PVN^CRH^ neurons (B) and statistical analysis of the colocalization of c‐Fos with CRH in each group (C) (*n* = 3–4). (D) Brain slice electrophysiology showing that CNO caused a rapid depolarization of mCherry^+^ PVN neurons in hM3Dq‐mCherry mice. (E) The quantification of membrane potential changes (*n* = 14 cells from 3 mice in each group). (F) Serum corticosterone levels of the mCherry and hM3Dq‐mCherry group (*n* = 7–8). (G) Serum ALT and AST levels in mice from each group (*n* = 6). (H) Representative images of TUNEL staining of liver tissues from each group. (I, J) Representative images and Suzuki score of H&E staining of liver tissues from each group (*n* = 9). (K) Relative expression of mRNAs for cytokines of *Tnf‐α*, *Il‐1β* and *Ccl2* (*n* = 7). (L) Serum concentrations of TNF‐α and IL‐1β (*n* = 6–7). Two‐tailed unpaired *t* test (C, F, J–L). Two‐tailed paired *t* test (E). One‐way ANOVA (G). ns, not significant; * *p* < 0.05; **p < 0.01; ****p < 0.0001.

To further investigate the role of CRH neurons in regulating HIRI, we employed chemogenetic manipulations to activate these neurons by injecting an adeno‐associated virus encoding Cre‐dependent expression of excitatory DREADDs, hM3Dq, into the PVN of *Crh‐cre* mice (Figure 5A). The result of immunostaining and brain slice electrophysiology showed that the PVN^CRH^ neurons were significantly activated by the administration of the DREADD ligand clozapine‐N‐oxide (CNO) (Figure 5B–E). Compared to controls, chemogenetic activation of PVN^CRH^ neurons resulted in a significant elevation of serum corticosterone level (Figure 5F). While no significant changes were observed between the two groups of mice in terms of total distance traveled within the open field and time spent in the central area (Figure S5A,B). Importantly, chemogenetic activation of PVN^CRH^ neurons significantly reduced plasma aminotransferase levels of HIRI mice (Figure 5G), which mimics the effects of ARS. We also found that necrosis and apoptosis of liver tissues were improved in the hM3Dq‐mCherry+HIRI group (Figure 5H–J). Moreover, the mRNA levels of the classical inflammatory factors, *Tnf‐α*, *Il‐1β* and *Ccl2*, and the protein content of IL‐1β in hM3Dq‐mCherry+HIRI mice were also decreased (Figure 5K,L).

### 3.6 Chemogenetic Inhibition of the PVN^CRH^ Neurons Reverses the Protective Effect of ARS on HIRI

**FIGURE 6:**
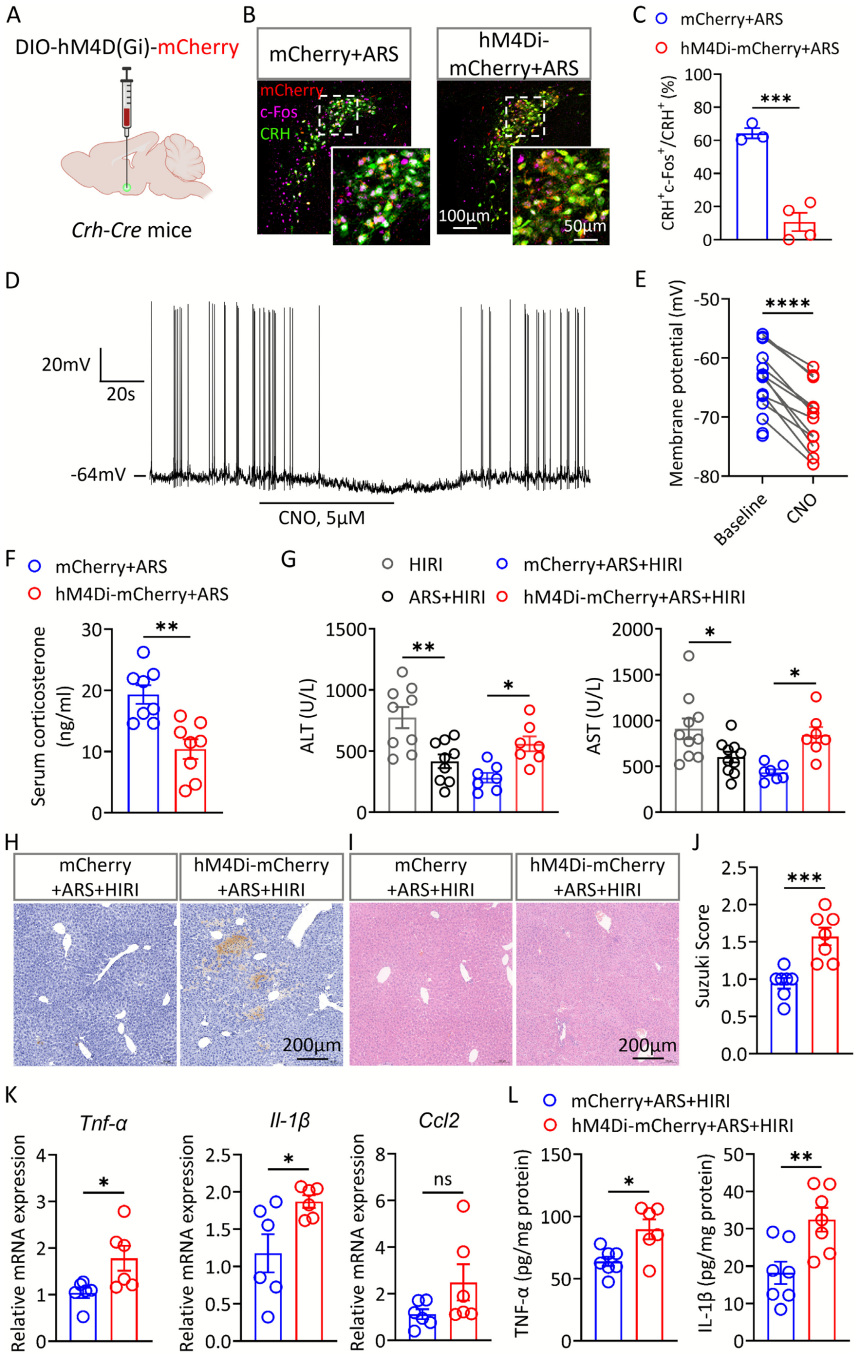
Chemogenetic inhibition of the PVN^CRH^ neurons reverses the protective effect of ARS on HIRI. (A) Paradigm of chemogenetic inhibition of PVN^CRH^ neurons. (B, C) Representative images showing the expression of mCherry or hM4Di‐mCherry and c‐Fos in the PVN^CRH^ neurons of mice under ARS (B) and the colocalization of c‐Fos with CRH in each group (C) (*n* = 3–4). (D) Representative trace of membrane potential hyperpolarization in PVN^CRH^ neurons induced by CNO administration. (E) The quantification of membrane potential changes (*n* = 14 cells from 3 mice in each group). (F) Serum corticosterone levels of the mCherry and hM3Dq‐mCherry group (*n* = 7–8). (G) Serum ALT and AST levels in mice from each group (*n* = 6–7). (H) Representative images of TUNEL staining of liver tissues from each group. (I, J) Representative images and Suzuki score of H&E staining of liver tissues from each group (*n* = 6–8). (K) Relative expression of mRNAs for cytokines of *Tnf‐α*, *Il‐1β* and *Ccl2* (*n* = 7–8). (L) Serum concentrations of TNF‐α and IL‐1β (*n* = 6–7). Two‐tailed unpaired *t* test (C, F, J–L). Two‐tailed paired *t* test (E). One‐way ANOVA (G). ns, not significant; * *p* < 0.05; **p < 0.01; ***p < 0.001; ****p < 0.0001.

We wondered whether inhibiting CRH neurons during restraint stress could reverse the protective effect of ARS on HIRI. We chemogenetically inhibited the activity of PVN^CRH^ neurons of *Crh‐*cre mice, and then performed ARS model and HIRI surgery. The effectiveness of the chemogenetic manipulations was confirmed by immunostaining and brain slice electrophysiology recording (Figure 6A–E). We found that chemogenetic inhibition of PVN^CRH^ neurons markedly reduced the serum corticosterone level elevated by ARS (Figure 6F), but had no significant effect on the total traveled distance or time spent in the central area during the OFT (Figure S6A,B). The result showed a significant increase in liver transaminase levels in hM4Di‐mCherry+ARS + HIRI group compared to the mCherry+ARS + HIRI group (Figure 6G), which implied that inhibition of PVN^CRH^ neurons could reverse the protective effect of acute stress on HIRI, consistent with the results of increased tissue necrosis, apoptosis and inflammatory response in the liver (Figure 6H–L).

## 4 Discussion

The relationship between stress and the development of a wide range of diseases has been demonstrated in studies over the past few decades. Chronic stress, in particular, has been recognized as a risk factor for a variety of psychiatric disorders, as well as affecting cardiovascular disease, cancer, and infectious diseases [26, 27, 28]. However, many studies have found that acute or low to moderate levels of stress (eustress) may have beneficial effects [7, 9]. In the present study, we tested the hypothesis that acute stress attenuates hepatic ischemia–reperfusion injury and found that corticosterone preconditioning or activation of CRH neurons in the PVN provided equivalent protection against HIRI in mice.

Stress modulates peripheral organ function through two primary pathways: activation of the HPA axis leading to the release of glucocorticoids (corticosterone in rodents) and activation of the SNS resulting in norepinephrine (NE) release. Meng et al. discovered that restraint stress induces increased androgen secretion in male mice. These androgens bind to androgen receptors on PVN^CRH^ neurons, activating the HPA axis and triggering corticosterone release, ultimately leading to immune cell apoptosis and thymic atrophy [29]. Liu et al. identified the hepatic sympathetic nerve signaling pathway as a critical downstream effector through which the PVN‐ventromedial hypothalamus (VMH)‐raphe pallidus nucleus (Rpa) multilevel neural circuit regulates the timely provision of glucose during the early phase of stress [19]. In this study, we showed that ARS did not increase hepatic NE levels. Furthermore, chemical sympathectomy with 6‐OHDA failed to diminish the ARS‐conferred protection against HIRI, suggesting the mechanism is independent of SNS involvement. In contrast, ARS markedly elevated serum corticosterone levels. Corticosterone pretreatment mimicked the protective effect of ARS, whereas pharmacological blockade of the GR completely abolished this effect. Together with previous evidence supporting the anti‐apoptotic effect of glucocorticoids in HIRI [30], as well as a negative correlation observed between hepatic GR expression and postoperative aspartate aminotransferase levels in an orthotopic liver transplantation cohort [31], these findings collectively support the conclusion that ARS attenuates HIRI primarily through HPA axis activation and subsequent corticosterone release, rather than through SNS‐mediated mechanisms.

The hypothalamic PVN acts as a central hub for stress integration and functions as a dual regulator of both the HPA axis and the sympathetic regulatory centers [19, 32]. Within the PVN, multiple neuronal subpopulations respond to stress, with CRH neurons representing the canonical mediators of HPA axis activation [25]. In this study, we demonstrated that ARS selectively activates PVN^CRH^ neurons. Chemogenetic activation of these neurons recapitulated the protective effect of ARS against HIRI, while their inhibition abolished ARS‐induced hepatoprotection, establishing a causal link and identifying PVN^CRH^ neurons as the central neural substrate underlying ARS‐mediated liver protection. These results align with previous reports showing that various forms of acute stress—such as footshock, restraint, fasting, and social defeat—rapidly activate PVN^CRH^ neurons, enhancing their firing rates and synaptic plasticity while elevating corticosterone levels [19, 32, 33, 34, 35]. Together, these findings support a general model in which rapid activation of PVN^CRH^ neurons drives HPA axis mobilization. It is worth noting, however, that different stress patterns may preferentially engage non‐CRH neuronal populations within the PVN, such as oxytocin and arginine vasopressin neurons [36, 37].

Extensive research has revealed that acute and chronic stress often exert opposing effects on the body. Acute stress typically engages short‐term adaptive mechanisms that facilitate rapid responses to external challenges, thereby frequently conferring protective benefits. In contrast, prolonged stress disrupts nervous system function and peripheral organ homeostasis, often leading to detrimental outcomes. In this study, we demonstrated that ARS confers significant protection against HIRI, aligning with the documented protective roles of acute stress in other organ systems. For instance, acute physical stress protects the heart from ischemia/reperfusion injury through activation of the SNS [38]. Similarly, short‐term stress preconditioning alleviates testicular ischemia–reperfusion injury via sympathetic‐mediated mechanisms [9]. Furthermore, ARS has been shown to activate medullary C1 neurons, thereby ameliorating renal ischemia–reperfusion injury through sympathetic—rather than vagal—pathways [7]. Conversely, chronic stress generally disrupts peripheral organ homeostasis. Our recent work revealed that chronic stress significantly suppresses liver regeneration capacity [14]. Additionally, repeated stress impairs the ability of the medial amygdala (MeA) to regulate blood glucose, resulting in disrupted hepatic glucose metabolism [39]. Moreover, a recent study indicates that chronic restraint stress induces persistent activation of neuroimmune pathways, elevating γ‐aminobutyric acid (GABA) and reducing acetylcholine (ACh) levels within the tumor microenvironment, which in turn promotes colorectal cancer progression [16]. Therefore, it is crucial to leverage the transient protective window offered by acute stress while preventing the establishment and persistence of chronic stress.

In future clinical practice, increased attention should be given to the assessment of preoperative stress levels in patients. Evaluating preoperative stress may aid in predicting surgical outcomes, particularly among individuals undergoing liver procedures, and could contribute to preventive strategies against HIRI. Moreover, for patients exhibiting significant or chronic stress, comprehensive interventions—including psychological support, pharmacotherapy, and neuromodulation—should be implemented to optimize overall surgical results.

## 5 Conclusion

In summary, our study demonstrates that the protective effect of ARS against HIRI in mice is mediated through the HPA axis rather than the SNS. The underlying mechanism involves ARS‐induced activation of PVN^CRH^ neurons, which drives the HPA axis to release corticosterone, thereby alleviating hepatic inflammation and ultimately attenuating HIRI. These findings provide novel targets and strategic insights for the perioperative prevention or mitigation of HIRI.

## Author Contributions

P.G. and LQ.Y. designed the experiments. XQ.L., P.G., D.Y., SY.W. and BS.W. performed the experiments and analyzed the data. XQ.L. and P.G. wrote the manuscript. P.G., LQ.Y., YF.J., WF.Y., L.Z., YY.Z., S.Z., YF.Z. and QH.Z. provided critical suggestions and revised the manuscript. P.G., LQ.Y., and XQ.L. completed the final review and submitted the manuscript. All authors contributed to the article and approved the submitted version.

## Funding

This study was supported by the National Natural Science Foundation of China (No. 82371517, 82270916, U23A20508, 82300725, 82371478), National Key Research and Development Program of China: Special Project for Modernization of Traditional Chinese Medicine—Young Scientist Project (No. 2025YFC3510700), the Fundamental Research Funds for the Central Universities (No. 25X010202804), Sanming Project of Medicine in Shenzhen (No. SZSM202311003), and Shanghai Engineering Research Center of Peri‐operative Organ Support and Function Preservation (No. 20DZ2254200).

## Ethics Statement

The animal experiments were approved by the Ethics Committee for Experimental Use of Animals of Shanghai Jiao Tong University School of Medicine (No. SYXK‐2013‐0050).

## Conflicts of Interest

The authors declare no conflicts of interest.

## Supporting information


**Figure S1:** (A) Representative track map of the open‐field test (OFT) in naive and ARS mice. (B) Mice from both groups showed no significant difference in the total distance traveled or the time spent in the center of the open field (*n* = 5–7 mice). (C) Representative images of TUNEL staining of liver tissues from each group. Two‐tailed unpaired *t* test (B). ns, not significant.
**Figure S2:** (A) Representative images of TUNEL staining of liver tissues from each group. (B) Relative expression of mRNAs for cytokines of *Ccl2* (*n* = 7–8). (C, D) Representative images and Suzuki score of H&E staining of liver tissues from each group (*n* = 7). (E) Relative expression of mRNAs for cytokines of *Tnf‐α*, *Il‐1β*, and *Ccl2* (*n* = 8–10). One‐way ANOVA (B). Two‐tailed unpaired *t* test (D and E). ns, not significant. *p < 0.05; **p < 0.01; ***p < 0.001.
**Figure S3:** Denervation of hepatic sympathetic nerves did not alter the effects of ARS on HIRI. (A) NE levels in liver tissue of Naive and ARS mice (*n* = 6). (B) Experimental outlines for ARS and HIRI modeling after 6‐OHDA administration. (C) NE levels in liver tissue of Vehicle+ARS and 6‐OHDA+ARS group mice (*n* = 8). (D) Serum ALT and AST levels in mice from each group (*n* = 9–10). (E, F) Representative images and Suzuki score of H&E staining of liver tissues from each group (*n* = 5–6). (G) Relative expression of mRNAs for cytokines of *Tnf‐α*, *Il‐1β*, and *Ccl2* (*n* = 8–9). Two‐tailed unpaired *t* test (A, C, D, F and G). ns, not significant; ****p < 0.0001.
**Figure S4:** The threshold current for inducing action potentials is lower in ARS mice (*n* = 18 cells from 3 mice in each group). Two‐tailed unpaired *t* test, ***p < 0.001.
**Figure S5:** Chemogenetic activation of PVN^CRH^ neurons has no significant impact on locomotion in mice during the OFT. (A) Representative track map of the OFT in mCherry and hM3Dq‐mCherry group mice. (B) Mice from both groups showed no significant difference in the total distance traveled or the time spent in the center of the open field (*n* = 6 mice for each group). Two‐tailed unpaired *t* test (B). ns, not significant.
**Figure S6:** Chemogenetic inhibition of PVN^CRH^ neurons has no significant impact on locomotion in ARS mice during the OFT. (A) Representative track map of the OFT in mCherry+ARS and hM4Di‐mCherry+ARS group mice. (B) Mice from both groups showed no significant difference in the total distance traveled or the time spent in the center of the open field (*n* = 6 mice for each group). Two‐tailed unpaired *t* test (B). ns, not significant.
**Table S1:** Primer sequences.

## Data Availability

The data that support the findings of this study are available from the corresponding author upon reasonable request.
