# Supplementary material for: Acute Stress Attenuates Hepatic Ischemia–Reperfusion Injury via Hypothalamic CRH Neuron‐Induced HPA Axis Activation
Source: CNS Neurosci Ther. 2026 Jan 14;32(1):e70749. doi: 10.1002/cns.70749 (PMC12802567; doi:10.1002/cns.70749)
Supplement: Supplementary file 1 — Figure S1: (A) Representative track map of the open‐field test (OFT) in naive and ARS mice. (B) Mice from both groups showed no significant difference in the total distance traveled or the time spent in the center of the open field (n = 5–7 mice). (C) Representative images of TUNEL staining of liver tissues from each group. Two‐tailed unpaired t test (B). ns, not significant. Figure S2: (A) Representative images of TUNEL staining of liver tissues from each group. (B) Relative expression of mRNAs for cytokines of Ccl2 (n = 7–8). (C, D) Representative images and Suzuki score of H&E staining of liver tissues from each group (n = 7). (E) Relative expression of mRNAs for cytokines of Tnf‐α, Il‐1β, and Ccl2 (n = 8–10). One‐way ANOVA (B). Two‐tailed unpaired t test (D and E). ns, not significant. *p < 0.05; **p < 0.01; ***p < 0.001. Figure S3: Denervation of hepatic sympathetic nerves did not alter the effects of ARS on HIRI. (A) NE levels in liver tissue of Naive and ARS mice (n = 6). (B) Experimental outlines for ARS and HIRI modeling after 6‐OHDA administration. (C) NE levels in liver tissue of Vehicle+ARS and 6‐OHDA+ARS group mice (n = 8). (D) Serum ALT and AST levels in mice from each group (n = 9–10). (E, F) Representative images and Suzuki score of H&E staining of liver tissues from each group (n = 5–6). (G) Relative expression of mRNAs for cytokines of Tnf‐α, Il‐1β, and Ccl2 (n = 8–9). Two‐tailed unpaired t test (A, C, D, F and G). ns, not significant; ****p < 0.0001. Figure S4: The threshold current for inducing action potentials is lower in ARS mice (n = 18 cells from 3 mice in each group). Two‐tailed unpaired t test, ***p < 0.001. Figure S5: Chemogenetic activation of PVNCRH neurons has no significant impact on locomotion in mice during the OFT. (A) Representative track map of the OFT in mCherry and hM3Dq‐mCherry group mice. (B) Mice from both groups showed no significant difference in the total distance traveled or the time spent in the center of the open field [file CNS-32-e70749-s001.docx]

**Supplementary Material**

**Acute Stress Attenuates Hepatic Ischemia-Reperfusion Injury via Hypothalamic CRH Neuron-Induced HPA Axis Activation**

Xiaoqi Lin^1,2,^^#^, Dan Yang^1,2,#^, Shuyang Wang^1,2,#^, Baoshan Wang^1,2^, Ling Zhu^1,2^, Yanyu Zhou^1,2^, Yifei Zhou^1,2^, Song Zhang^1,2^, Qionghui Zhan^1,2^, Yingfu Jiao^1,2^, Weifeng Yu^1,2^, Liqun Yang^1,2,3,4,*^ Po Gao^1,2,5,*^

^1^Department of Anesthesiology, Renji Hospital, Shanghai Jiao Tong University School of Medicine, Shanghai, 200127, China

^2^Key Laboratory of Anesthesiology (Shanghai Jiao Tong University), Ministry of Education, Shanghai, China.

^3^Department of Pain, The First Affiliated Hospital of Wenzhou Medical University, Wenzhou, Zhejiang, China.

^4^Oujiang Laboratory (Zhejiang Lab for Regenerative Medicine, Vision and Brain Health), Wenzhou Medical University, Wenzhou, Zhejiang, China

^5^Department of Anesthesiology, The Fourth Affiliated Hospital of Anhui Medical University, Chaohu, Anhui, China

^#^These authors contributed equally to this work.

*Correspondence: [gaopo0908@163.com](mailto:gaopo0908@163.com) (P.G.), [lqyang72721@126.com](mailto:lqyang72721@126.com) (L.Y.)

**Supplementary Figures**


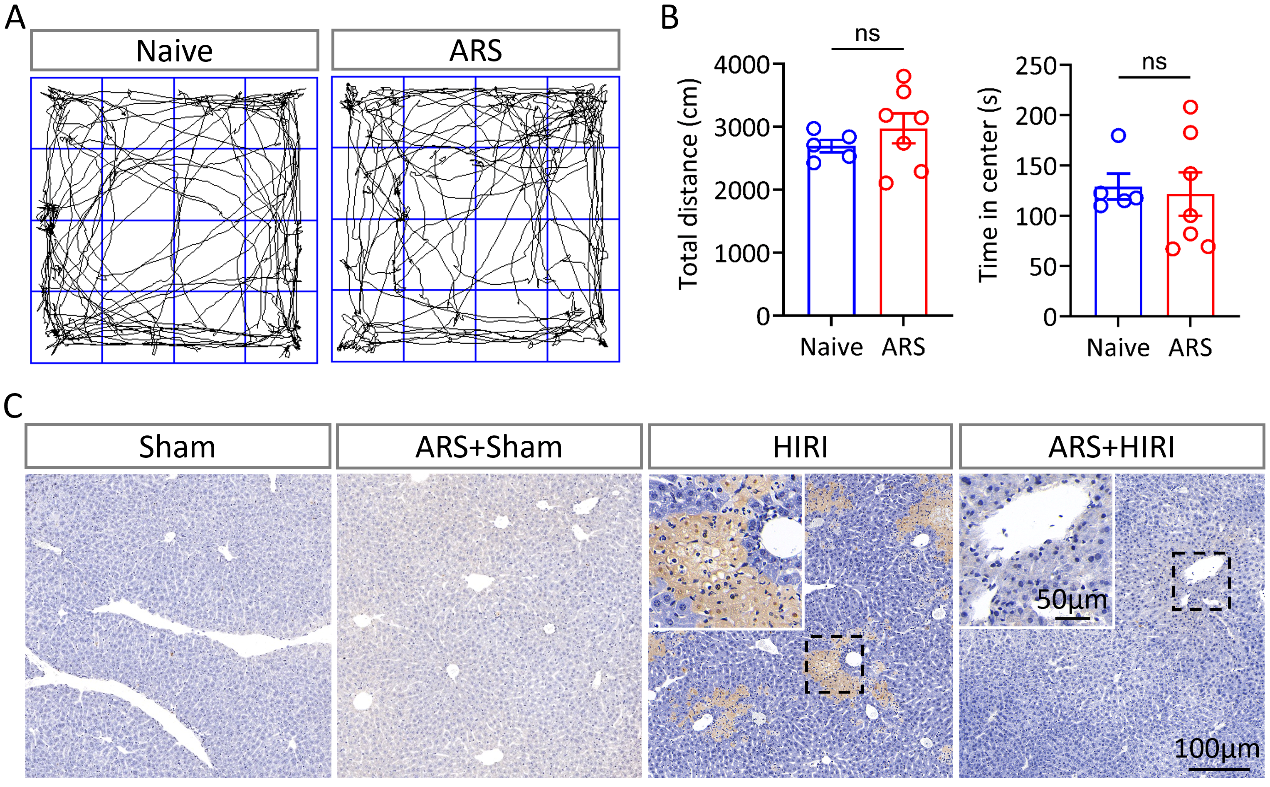


**Figure S1. (A)** Representative track map of the open-field test (OFT) in naive and ARS mice. **(B)** Mice from both groups showed no significant difference in the total distance traveled or the time spent in the center of the open field (*n* = 5-7 mice). (**C**) Representative images of TUNEL staining of liver tissues from each group. Two-tailed unpaired *t* test (**B**). ns, not significant.


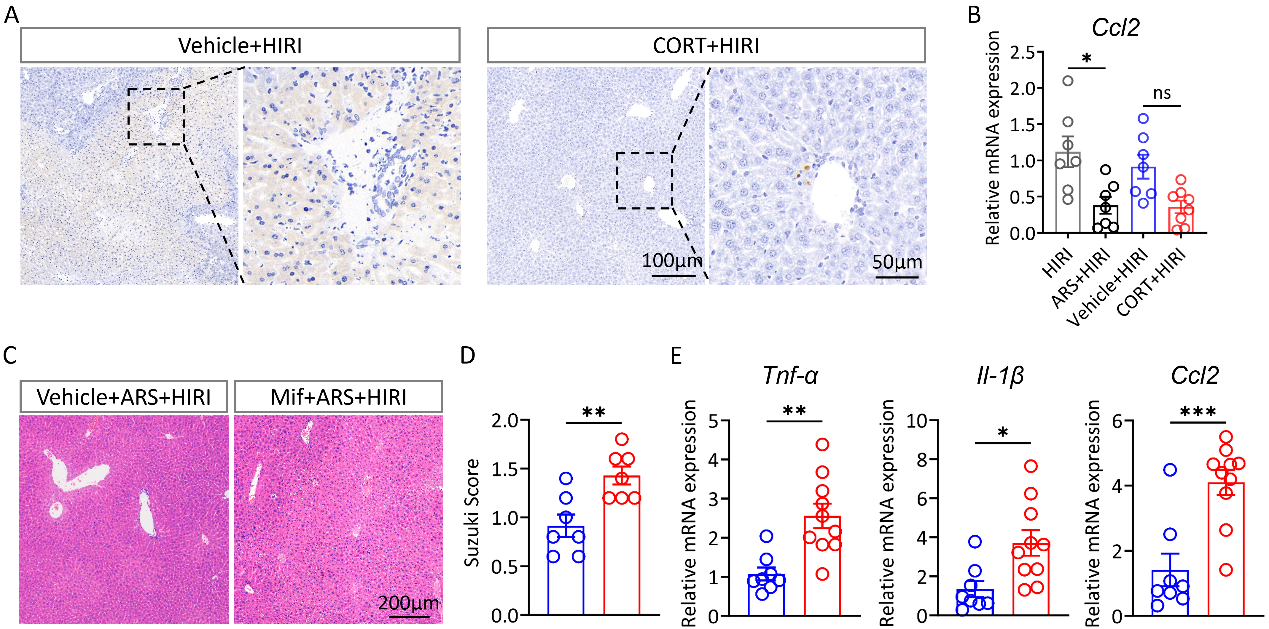


**Figure S2. (A)** Representative images of TUNEL staining of liver tissues from each group. **(B)** Relative expression of mRNAs for cytokines of *Ccl2* (*n* = 7-8). **(C-D)** Representative images and Suzuki score of H&E staining of liver tissues from each group (n = 7). **(E)** Relative expression of mRNAs for cytokines of *Tnf-α*, *Il-1β* and *Ccl2* (n = 8-10). One-way ANOVA (**B**). Two-tailed unpaired *t* test (**D** and **E**). ns, not significant. * *p* < 0.05; ** *p* < 0.01; *** *p* < 0.001.


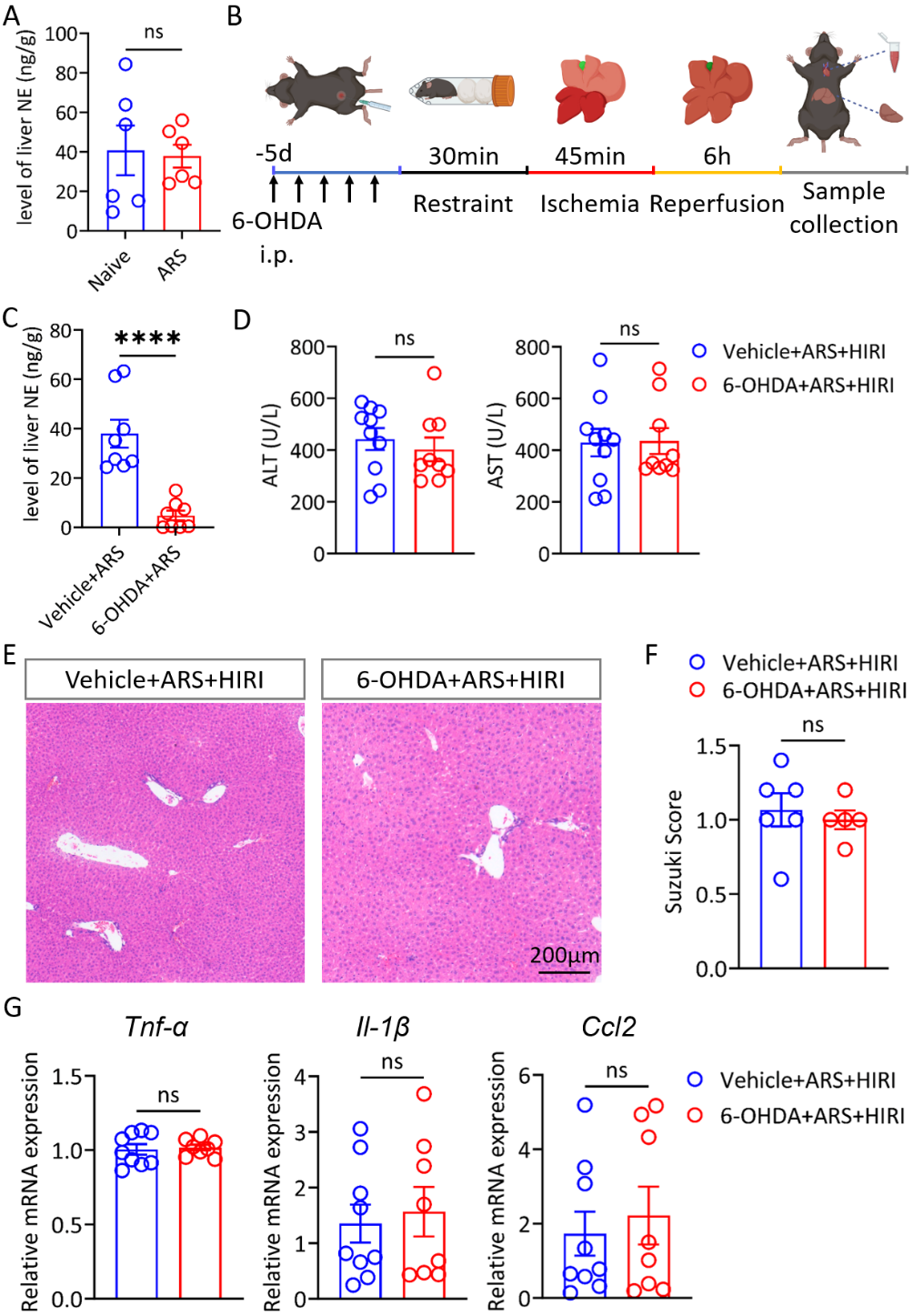


**Figure S3.** Denervation of hepatic sympathetic nerves did not alter the effects of ARS on HIRI. **(A)** NE levels in liver tissue of Naive and ARS mice (*n* = 6). **(B)** Experimental outlines for ARS and HIRI modeling after 6-OHDA administration. **(C)** NE levels in liver tissue of Vehicle+ARS and 6-OHDA+ARS group mice (*n* = 8). **(D)** Serum ALT and AST levels in mice from each group (*n* = 9-10). **(E-F)** Representative images and Suzuki score of H&E staining of liver tissues from each group (*n* = 5-6). **(G)** Relative expression of mRNAs for cytokines of *Tnf-α*, *Il-1β* and *Ccl2* (*n* = 8-9)*.* Two-tailed unpaired *t* test (**A**, **C**, **D**, **F** and **G**). ns, not significant; **** *p* < 0.0001.


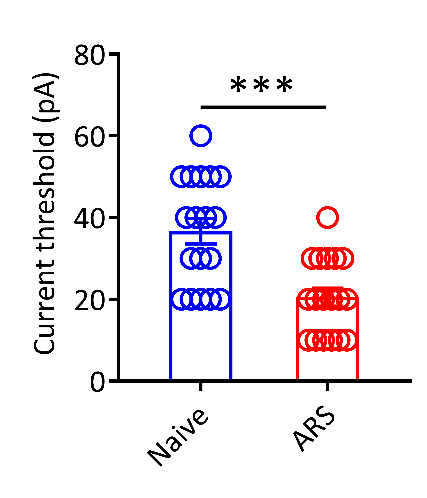


**Figure S4.** The threshold current for inducing action potentials is lower in ARS mice (n = 18 cells from 3 mice in each group). Two-tailed unpaired *t* test, *** *p* < 0.001.


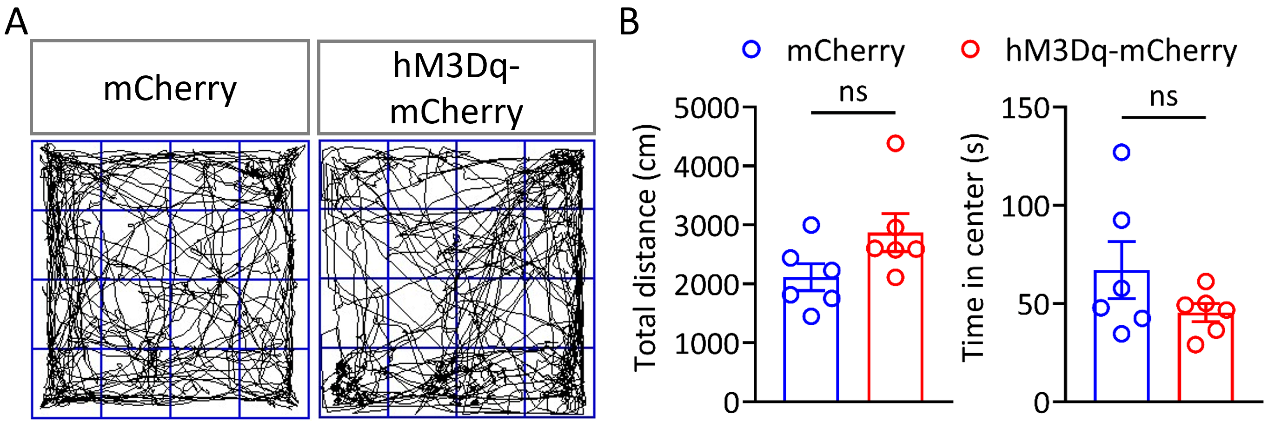


**Figure S5.** Chemogenetic activation of PVN^CRH^ neurons has no significant impact on locomotion in mice during the OFT. **(A)** Representative track map of the OFT in mCherry and hM3Dq-mCherry group mice. **(B)** Mice from both groups showed no significant difference in the total distance traveled or the time spent in the center of the open field (*n* = 6 mice for each group). Two-tailed unpaired *t* test (**B**). ns, not significant.


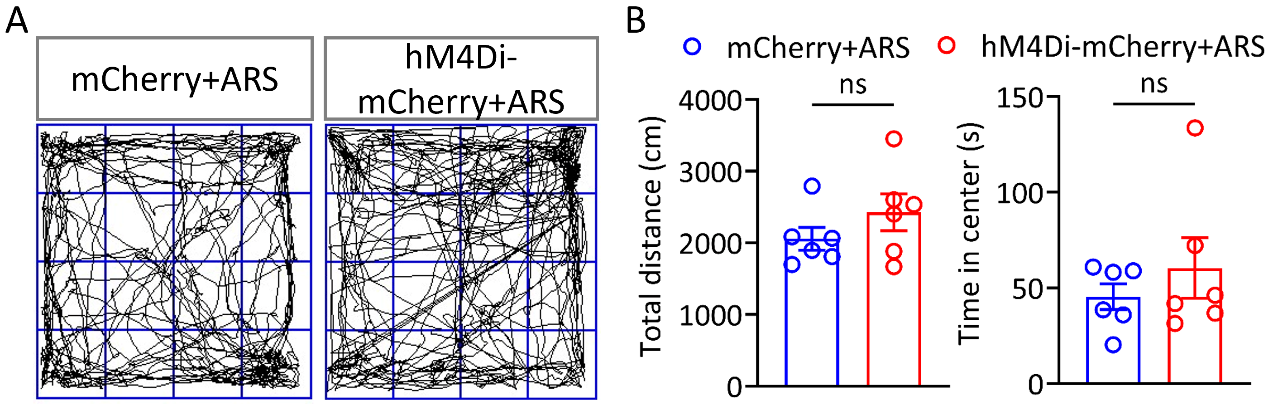


**Figure S6.** Chemogenetic inhibition of PVN^CRH^ neurons has no significant impact on locomotion in ARS mice during the OFT. **(A)** Representative track map of the OFT in mCherry+ARS and hM4Di-mCherry+ARS group mice. **(B)** Mice from both groups showed no significant difference in the total distance traveled or the time spent in the center of the open field (*n* = 6 mice for each group). Two-tailed unpaired *t* test (**B**). ns, not significant.

**Supplementary Table**

**Supplementary table 1.** Primer sequences.

| **Gene** | **Forward primer** | **Reverse primer** |
| --- | --- | --- |
| *Gapdh* | AGGTCGGTGTGAACGGATTTG | TGTAGACCATGTAGTTGAGGTCA |
| *Tnf-α* | AGCCGATGGGTTGTACCTTG | ATAGCAAATCGGCTGACGGT |
| *Il-1β* | GCAACTGTTCCTGAACTCAACT | ATCTTTTGGGGTCCGTCAACT |
| *Ccl2* | TTAAAAACCTGGATCGGAACCAA | GCATTAGCTTCAGATTTACGGGT |
